# Genotype and Phenotype Interaction between *OsWKRYq6* and BLB after *Xanthomonas oryzae* pv. *Oryzae* Inoculation in the Field

**DOI:** 10.3390/plants11030287

**Published:** 2022-01-21

**Authors:** Xiao-Xuan Du, Jae-Ryoung Park, Xiao-Han Wang, Rahmatullah Jan, Gang-Seob Lee, Kyung-Min Kim

**Affiliations:** 1 Biosafety Division, National Academy of Agricultural Science, Rural Development Administration, Jeonju 54874, Korea; haobingshuaike@hotmail.com; 2 Coastal Agriculture Research Institute, Kyungpook National University, Daegu 41566, Korea; 3 Division of Plant Biosciences, School of Applied Biosciences, College of Agriculture and Life Science, Kyungpook National University, Daegu 41566, Korea; icd92@naver.com (J.-R.P.); rehmatbot@yahoo.com (R.J.); 4 Department of Crop Breeding, National Institute of Crop Science, Rural Development Administration, Wanju 55365, Korea; 5 National Agrobiodiversity Center, National Institute of Agricultural Sciences, Rural Development Administration, Jeonju 54875, Korea; wang@knu.ac.kr

**Keywords:** bacterial leaf blight, quantitative trait locus, rice, resistant, field

## Abstract

Bacterial leaf blight (BLB) is an important and devastating rice disease caused by the pathogen *Xanthomonas oryzae* pv. *Oryzae* (*Xoo*). In particular, in recent years, the occurrence of abnormal climate and warming phenomena has produced a good environment for the occurrence of BLB, and the rice yield due to the occurrence of BLB continues to decrease. Currently, molecular breeding is applied by searching for resistant genes to development of BLB resistance cultivar. In addition, there are many methods for screening resistant genes, and among them, phenotype analysis in the field and applied research is rarely conducted. Due to recent rapid climate change, BLB is a major problem that has a more serious negative effect on rice yield. Therefore, we suggest *OsWRKYq6* to be effectively used for breeding BLB-resistant cultivars by screening BLB-resistant genes. In this study, the BLB-resistant gene was screened using the lesion length, which most definitely changes to the phenotype when *Xoo* is infected. *OsWRKYq6* was finally selected as a BLB resistance gene by analyzing the phenotype and genotype after inoculating *Xoo* in 120 Cheongcheong/Nagdong double haploid (CNDH) lines in the field. After *Xoo* inoculation, lesion length and yield were investigated, and 120 CNDH lines were divided from BLB-resistant and susceptible lines. Moreover, when the transcription level of *OsWRKYq6* was analyzed in the resistant and susceptible lines after *Xoo* inoculation in the field, the expression level was regulated to a high level in the resistant line. In this study, we propose *OsWRKYq6* as a transcription factor involved in BLB resistance. Currently, the differentiation of various races is proceeding rapidly due to rapid climate change. In addition, screening of transcription factor genes involved in BLB resistance in the field can be effectively applied to molecular breeding to develop resistant cultivars in preparation for rapid climate change.

## 1. Introduction

FAO assesses that globally, 45 countries, 34 of which are in Africa, continue to be in need of external assistance for food. Conflicts, weather events, and pests remain critical factors underpinning the high levels of severe food insecurity. World rice production in 2020 is now predicted to reach an all-time high of 508.4 million tons, 1.5 percent above the 2019 reduced level but marginally down from the previous month’s expectations. At the same time, world rice stocks at the close of 2020/21 are pegged at 181.0 million tons, down 0.4 percent from their opening levels and 1.0 million tons below last month’s expectations. The monthly revision primarily reflects an anticipated decline in reserves in India, owing to improved export prospects for the county [1].

Rice is a central staple food crop of most of the world’s population and is among the three most important food crops, with maize and wheat being the other two. More than 3.5 billion people use rice as their staple food, which translates to at least half of the people worldwide [2,3]. Rice is cultivated in almost all over the world due to its adaptable nature under various environmental conditions and is regarded as a strategic crop for food security [4]. However, susceptibility of rice to different diseases and insects is a major problem that may lead to a reduction of rice yield in the near future. Previous reports show that 25% of the annual yield loss is caused by diseases and insects, such as stem borer insects that are most destructive, whereas rice blast caused by the fungus *Pyricularia oryzae* and bacterial blight caused by *Xanthomonas oryzae* pv. *oryzae* (*Xoo*) are two of the most dangerous and common diseases in rice production [5].

Bacterial leaf blight (BLB) caused by *Xoo* is one of the key devastating diseases in rice farming, mostly in tropical Asia [6]. *Xoo* is a vascular pathogen that enters rice leaves through wounds and hydathodes. It enters into xylem vessels after initial multiplication in the epithem, and it further multiplies in xylem tissue, spreads all throughout the leaves, and blocks water transport [7]. BLB was reported for the first time in Japan during 1884–1885, which then spread and was reported in other rice-growing countries [2]. *Xoo*, being the causative agent of BLB, causes a severe loss of rice yield, and the disease it brings is broadly prevalent among diverse varieties of rice worldwide [8]. Due to climatic changes, the increasing temperature increases rice’s susceptibility to *Xoo* and also provides favorable conditions for the growth of other pathogens, hence creating considerable challenges for BLB management [9,10,11]. A previous report showed that rice was severely affected by BLB due to heavy rainfall in tropical areas and particularly in Asia [12]. Similarly, the Mekong Delta is the major rice-producing area of Vietnam, but it is highly vulnerable to climate change, which would therefore make BLB more destructive [13]. In Pakistan, an alarming increase of BLB occurrence was reported in Basmati rice-growing areas [14]. *Xoo* causes BLB at different rice growth stages and is evident by either leaf blight or “Kresek” (acute wilting of young plants) symptoms. It mostly enters rice through leaf wounds or water pores. As the water pores are usually located at the margins of the upper parts of the leaf, the lesions with wavy margins start from the tip of the rice leaf and enlarge in size, turn yellow, and finally cause death of the plant [14].

The lesion caused by *Xoo*, expands from the leaf tip toward the base, and the symp-toms typically appear after a few days in the form of dead v-shaped yellow parts at the leaf tip, water-soaked area of the leaf, as well as the green leaf area at the base. Furthermore, the dead area and water-soaked area of the leaf develop lesions that extend toward the leaf base, thereby covering the whole leaf and sometimes extending toward the leaf sheath [15]. Different disease management strategies have been previously implemented to overcome yield losses and avoid disease epidemics, but the application of chemicals has been un-successful because pathogens have different sensitivity levels to the applied chemicals. The use of antibiotics is a strategy to control BLB in rice, but it has limitations due to the toxic chemical residues [16].

F_2_ line, near isogenic lines (NILs), recombinant inbred lines (RILs), and double haploid (DH) populations are used for genetic mapping of plants. The F_2_ line has a very wide range of high heterozygosity, but it is difficult to obtain DNA samples repeatedly. In order for NILs to be cultivated, it is necessary to repetitively carry out backcross, and in rice, since RILs require a generation progress of at least F_6_ even when using the single seed descent method [17,18], it takes a large amount of time and effort to create RILs. If a double haploid line created through anther culture is used in the genetic map construct, compared to the NIL or RIL line, the effort and cost used to completely fixation the characteristics can be saved. The CNDH (Cheongcheong/Nagdong double haploid) line is a group developed through anther culture of F_1_, which is a cross between Cheongcheong and Nagdong. In addition, CNDH line has been developed in the field every year at Kyungpook National University since 2010, and since it has a line with various traits, it is used for functional analysis of new genes.

In the current research on BLB resistance genes, many scientists have adopted map-based cloning technology, which has been successfully employed for characterizing dominant BLB resistance genes such as *Xa21*, *Xa1*, *Xa26*, and *Xa27*, leading to a generally accepted model of dominant gene-mediated disease resistance. Currently, molecular breeding is an emerging safe strategy to control pathogen invasion in different crops. Therefore, evaluating *Xoo*-resistant genes is necessary to develop BLB-resistant rice cultivars. This strategy is a cost effective and environment-friendly approach to control this serious threat. Among molecular breeding techniques, quantitative trait loci (QTL) analysis is an advance technique to measure several genes responsible for a specific trait. Numerous plant traits, such as yield, quality, and susceptibility or resistance to diseases, are controlled by several genes called quantitative traits. These traits vary among individuals, and the genetic variations of quantitative traits are controlled by QTLs that are used to identify the regions responsible for a specific trait. Similarly, the aim of the present study was to investigate the QTLs responsible for *Xoo* resistance and to determine the specific gene involved in resistance development.

## 2. Results

### 2.1. Phenotype Evaluation

**Figure 1:**
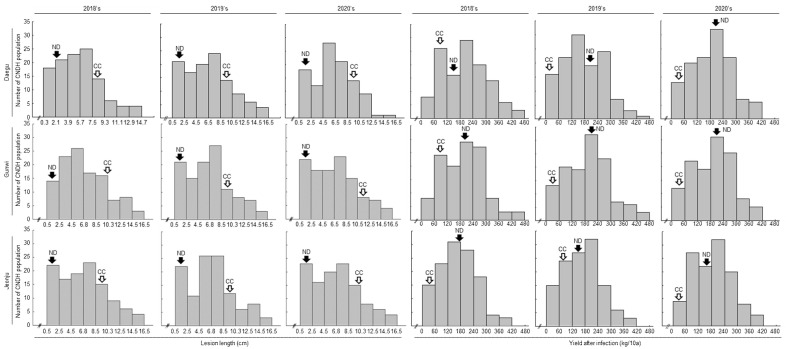
Distribution of lesion length and yield data in the CNDH line after the 14-day inoculation of *Xoo* K3 strain in an open field for 3 years. ND, Nagdong; CC, Cheongcheong.

The lesion length of each inoculated leaf was calculated after 14 days when the lesion length became prominent, and the data were recorded for QTL analysis. The phenotypic evaluation of the lesion length of the CNDH lines are represented in Figure S1. The frequency distribution of lesion length and yield data of both the years showed that Nagdong was resistant to *Xoo* while Cheongcheong was susceptible (Figure 1). Furthermore, we distributed the CNDH lines on the basis of lesion length and yield data. Therefore, in resistance lines, the shorter the lesion length, the lower the yield loss. Conversely, in susceptible lines, the longer the lesion length, the higher the yield loss. In 2018, 2019, and 2020, lesion length and yield were investigated after inoculation of *Xoo* into a 120 CNDH line. In each year, the lesion length and yield of the 120 CNDH line were investigated in three regions, Daegu, Gunwi, and Jeonju (Table S1). In Daegu in 2018, the lesion length in the range of 0.3–2.1 cm was the shortest, and 18 out of the 120 CNDH line belonged to this group. In the group with the longest lesion length, lesion lengths in the range of 12.9–14.7 cm were investigated, and four lines were included. In addition, the yield after *Xoo* inoculation was the least at 0–60 kg/10a, eight lines belonged to the group, and the highest yield range after *Xoo* inoculation was 420–480 kg/10a. There were three lines in the 420–480 kg/10a group. In Gunwi, the lesion length ranged from 0.5 to 2.5 cm in the group with the smallest length, and 14 CNDH lines were included. Conversely, the group with the longest lesion length had a lesion length ranging from 14.5 to 16.5 cm and included three CNDH lines. For yield, 0–60 kg/10a was the least, and 420–480 kg/10a was the most. These belong to 16 lines and 1 line, respectively. In Jeonju, the range of 0.5–2.5 cm lesion length was the smallest, and the range of 14.5–16.5 cm lesion length was the largest. In addition, yield was 0–60 kg/10a, being the smallest value, and 420–480 kg/10a was the largest yield range. In Daegu in 2019, the lesion length in the range of 0.5–2.5 cm was the shortest, and 21 of the 120 CNDH lines belonged to this group. In the group with the longest lesion length, lesion lengths ranging from 14.5 to 16.5 cm were investigated, and four lines were included. In addition, the yield after *Xoo* inoculation was the least at 0–60 kg/10a, eight lines belonged to the group, and the highest yield range after *Xoo* inoculation was 420–480 kg/10a. There are three lines in the 420–480 kg/10a group. In Gunwi, the lesion length ranged from 0.5 to 2.5 cm in the group with the smallest length, and 22 CNDH lines were included. Conversely, the group with the longest lesion length had a lesion length ranging from 14.5 to 16.5 cm and included four CNDH lines. For yield, 0–60 kg/10a was the least, and 420–480 kg/10a was the most. These belonged to 13 lines and 3 lines, respectively. In Jeonju, the range of 0.5–2.5 cm lesion length was the smallest, and the range of 14.5–16.5 cm lesion length was the largest. In addition, yield was 0–60 kg/10a and was the smallest value, and 420–480 kg/10a was the largest yield range. In Daegu in 2020, the lesion length in the range of 0.5–2.5 cm was the shortest, and 18 of the 120 CNDH lines belonged to this group. In the group with the longest lesion length, lesion lengths ranging from 14.5 to 16.5 cm were investigated, and one line belonged. In addition, after *Xoo* inoculation, the yield was the least at 0–60 kg/10a, 15 lines belonged to the group, and the highest yield range after *Xoo* inoculation was 360–420 kg/10a. There were three lines in the 360–420 kg/10a group. In Gunwi, the lesion length ranged from 0.5 to 2.5 cm to the group with the smallest length, and 22 CNDH lines were included. Conversely, the group with the longest lesion length had a lesion length ranging from 14.5 to 16.5 cm and included three CNDH lines. For yield, 0–60 kg/10a was the least, and 360–420 kg/10a was the most. These belonged to 15 lines and 3 lines, respectively. In Jeonju, the range of 0.5–2.5 cm lesion length was the smallest, and the range of 14.5–16.5 cm lesion length was the largest. In addition, yield was 0–60 kg/10a in the smallest value, and 360–420 kg/10a was the largest yield range.

### 2.2. Correlation Analysis of Yield and Lesion Length

**Figure 2:**
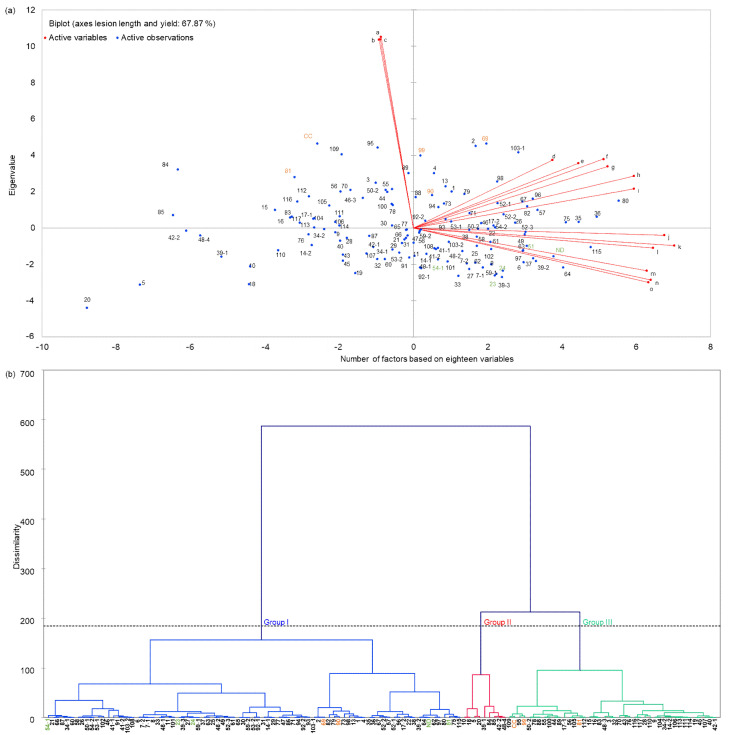
Statistical analysis results of yield, 1000 grain weight, and lesion length data of CNDH line for three years by PCA (principal component analysis) and AHC (agglomerative hierarchical clustering). (**a**) This figure is a two-dimensional result figure of PCA (principal component analysis). The lesion length and yield after infection explained 68.48% of variance. The figure shows the correlation lesion length, yield, and 1000 grain weight of CNDH line before and after inoculation over the past three years and their respective relationships and trends with the length of infected leaves. Numbers refer to the CNDH line numbers. (a) Infected lesion length 2018, (b) infected lesion length 2019, (c) infected lesion length 2020, (d) control 1000 grain weight 2018, (e) control 1000 grain weight 2019, (f) control 1000 grain weight 2020, (g) control yield 2018, (h) control yield 2019, (i) control yield 2020, (j) infected yield 2018, (k) infected yield 2019, (m) infected yield 2020, (n) infected 1000 grain weight 2018, (l) infected 1000 grain weight 2019, (o) infected 1000 grain weight 2020. (**b**) The chart below is the dendrogram of AHC (agglomerative hierarchical clustering) analysis. It represents how the algorithm works to group the observations, then the subgroups of observations. As is shown, the algorithm successfully grouped all the observations. This phylogenetic tree used combined lesion length and yield data. The dotted line represents the automatic truncation. Group I contains BLB resistant lines, Group II contains BLB moderate lines, and Group III contains BLB susceptible lines. The number on the X-axis means the CNDH line number. BLB resistance line is marked in green, and susceptible line is marked in orange. ND, Nagdong; CC, Cheongcheong.

In the present research, we evaluated the correlation between rice yield and lesion length in rice leaves after inoculation on the basis of the three-year field data of CNDH lines (Figure 2a, Table S2). Through statistical analysis, we found that as the length of rice leaf infection increased, the corresponding rice yield and 1000 grain weight decreased. Lesion length and yield had a negative correlation. Moreover, lesion length and 1000 grain weight were negatively correlated. However, yield and 1000 grain weight were positively correlated. Simultaneously, this also illustrates the direct relationship between the lesion length of the leaves of the CNDH lines after *Xoo* inoculation, as well as the yield reduction rate. In addition, the phylogenetic tree was analyzed using yield and lesion length data, and the CNDH lines were divided into three groups (Figure 2b). In Group I, both resistant and susceptible lines were present against BLB. However, the genetic distance for each line was very high. Group III was mostly BLB susceptible lines. Moreover, in Group II, moderative lines for BLB were distributed.

### 2.3. QTLs Analysis for Leaf Lesion Length

When using lesion length, we detected 13 QTLs, and when using yield data, we detected 5 QTLs. qll6, qll6-1, qll6-2, qll6-3, qll6-4, qll6-5, qYd6, and qYd6-1 were detected in RM20092-RM20176 on chromosome 6. They all had an LOD score of 3.0 or higher, and all were derived from the Cheongcheong allele. qll8 and qll8-2 were detected in RM1345-RM17699 of chromosome 8. The LOD scores were 3.09 and 3.20, respectively, and both were derived from the Cheongcheong allele. qll8-1, qYd8, qYd8-1, and qYd8-2 were detected in RM23178-RM23230 of chromosome 8, and all had LOD score of 3.0 or higher, and all were derived from the Cheongcheong allele. qll11, qll11-1, qll11-2, and qll11-3 are regions detected with LOD score of 3.0 or higher in RM26981-RM27161 of chromosome 11. These were all derived from the Cheongcheong allele (Figure S2, Table S3). When QTL mapping was analyzed, RM20092-RM20176 of chromosome 6 was a region detected with an LOD score of 3.0 or higher for 3 years.

### 2.4. Target Region Identification

In the three-year data analysis of the CNDH line against BLB leaf lesion length, five loci in three different chromosomal regions were identified as having phenotypic variations less than 2%. All the identified regions showed positive additive effect, which showed that the alleles were from the resistant parents and were BLB-resistant. We used NCBI, RAP DB, RICE PRO, and JUST BIO databases to obtain specific information about these areas. As a result, we obtained relevant information on 5990 genes and made a simple classification (Figure S3).

### 2.5. Physical Mapping Associated with Lesion Length

**Figure 3:**
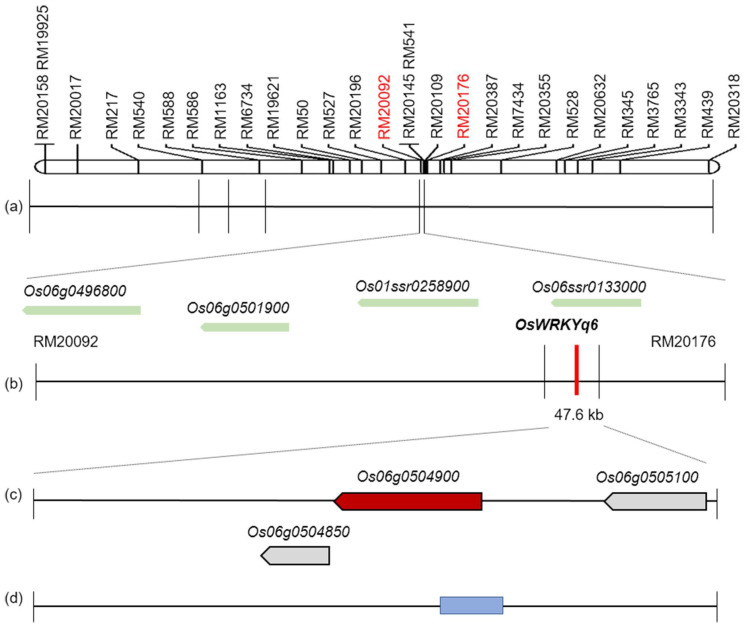
Physical map on the lesion length, relative to the QTL analysis at chromosome 6. (**a**) The chromosomal location of the QTL associated with lesion length of the CNDH genetic map. (**b**) The region of target area from RM20092 to RM20176. (**c**) The target genes (red) screened in the interval from RM20092 to RM20176. (**d**) The ORFs including redox signaling and hormone signaling. The blue diamond indicates the target gene of *OsWRKYq6*, which was found to improve BLB resistance.

As the same time, we found that the region between the target region marker RM20092 and the marker RM20176 of chromosome 6 overlapped in the two consecutive years of data. According to the results of QTL analysis, we found that there was the highest probability of the related BLB resistance gene we needed in the target range of chromosome 6. Therefore, we screened out 28 transcription factor gene involved in BLB resistance with leaf lesion length data in this interval through the NCBI, RAP DB, RICE PRO, and JUST BIO databases and classified them by function (Table S4). Simultaneously, we conducted a second screening of these 28 transcription factor genes involved in BLB resistance, selected *OsWRKYq6* as the target gene, and generated the related physical map (Figure 3).

### 2.6. Gene Information Analysis

**Figure 4:**
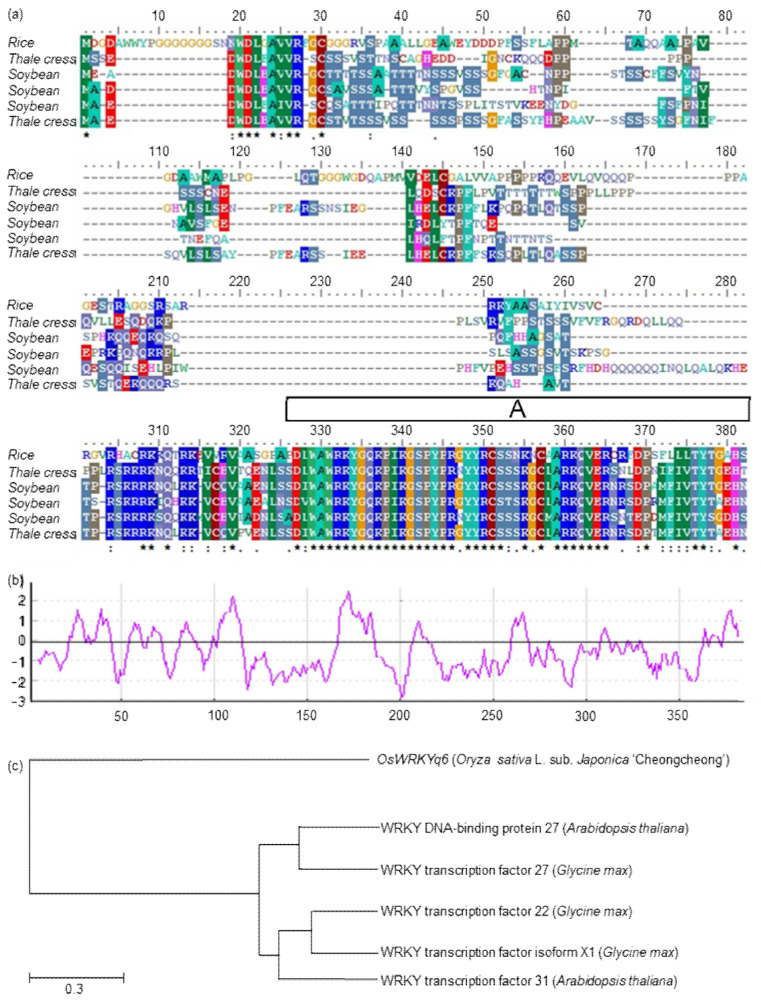
Characterization and identification of *OsWKRYq6* similar to WRKY proteins. Similar protein sequences were analyzed using the NCBI database, and the same amino acids were labeled. (**a**) Multiple sequence comparison analysis was performed using the BioEdit program and the constraint-based multiple alignment. Motifs were detected using the MotifFinder soft. The “A” is labeled as WRKY at amino acids 326–382; (**b**) is the hydrophobicity plot for the *OsWRKYq6* protein, and the upper and lower cutoffs are marked. Regions with values above 0 are hydrophobic in character. X-axis: amino acid position, Y-axis: mean hydrophobicity. (**c**) The phylogenetic tree analysis of similar WRKY proteins from plants and organisms that was constructed using the UPGMA method.

Among these genes, we found a WRKY gene that has never been reported to be resistant to BLB and generated the related physical map for this target gene (Figure 3). The gene of WRKY family is highly associated with plant defense. We characterized and identified *Os06g0504900*, which is similar to the WRKY protein. We analyzed similar protein sequences in reference to the NCBI database and marked the same amino acids (Figure 4a). Multi-sequence comparative analysis was performed using the BioEdit program. In addition, most of the *WRKY* gene and homology regions were hydrophobic regions (Figure 4b). We named it *OsWRKYq6*. *OsWRKYq6* is not identical to the gene sequence related to the existing WRKY transcription factor but has a similar sequence (Figure 4c). In addition, the sequence of *OsWRKYq6* is the same in resistant and susceptible lines against BLB (data not shown). Therefore, it was named *OsWRKYq6* because it was mapped on chromosome 6.

### 2.7. Results of Quantitative RT-PCR Analyses

**Figure 5:**
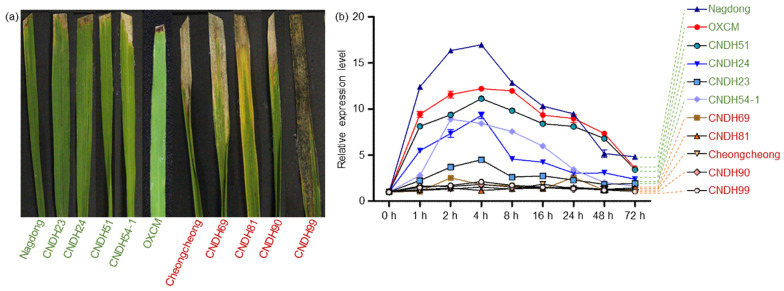
Phenotype and genotype evaluation after *Xoo* inoculation. (**a**) Six groups (Nagdong, CNDH23, CNDH24, CNDH51, CNDH54-1, OXCM) of BLB-resistant lines and five groups (Cheongcheong, CNDH69, CNDH81, CNDH90, and CNDH99) of BLB-susceptible lines were selected from the field. (**b**) Quantitative RT-PCR analysis result of the *OsWRKYq6* in the BLB-resistant and -susceptible lines in the CNDH lines. *OsWRKYq6* were highly expressed in BLB-resistant lines.

After completing the relevant database analysis, we verified whether the *OsWRKYq6* gene is a BLB-resistant transcription factor through a control group experiment. One group was the high-resistance group, and the other was the weak resistance group. The male and female parents (Cheongcheong and Nagdong) and BLB resistant line (OXCM), which was provided by Jan et al. [19], were simultaneously added for infection test. *OsWRKYq6* was amplified in all lines (Figure S4). The results of this analysis showed that *OsWRKYq6* is indeed the main gene that has resistance against the K3 strain of *X. oryzae* pv. *Oryzae*. (Figure 5). At 72 h after *Xoo* inoculation, only the leaves of the plants showing the average lesion length in each plant were selected, as shown in Figure 5a. After *Xoo* infection in the BLB-resistant line and BLB-susceptible line, leaves were sampled at 0 h, 1 h, 2 h, 4 h, 8 h, 16 h, 24 h, 48 h, and 72 h to analyze the expression level of *OsWRKYq6*. *OsWRKYq6* was highly expressed at 2h or 4h after *Xoo* infection in the BLB-resistant line. Moreover, in the BLB susceptible line, the expression was very weak.

## 3. Discussion

In order for the yield loss to be controlled in agriculture, it is essential to control the diseases that are caused by several pathogens. BLB caused by *Xoo* is a serious and devastating disease that is widely spread in rice-growing areas in the world. Until recently, approximately 40 BLB-resistant genes have been known and used in the breeding system designed to resist BLB infection [20]. Given the comparatively common breakdown of BLB resistance associated with the incidence of a new BLB pathotype or change of *Xoo* strain, it is essential to detect new resistance genes and associate these with known R-genes to develop strong and sustainable resistant lines. In the present study, we phenotypically evaluated the BLB lesion length caused by *Xoo* (Figure S1).

QTL is a technology that can best analyze the interaction between genotype and phenotype [21,22]. In this research, DH (double haploid) line was used for QTL mapping of transcription factor gene involved in BLB resistance. For QTL mapping, the NILs (near isogenic lines), RILs (recombinant inbred lines), and DH lines are mainly used. The DH line has the advantage of doubling the chromosomes through colchicine treatment of haploids, thus obtaining homo individuals in one generation [23]. However, even if the RILs group performs SSD (single seed descent), it is necessary to make progress by at least six generations [24], and the NILs group must repeatedly perform back crossing [25]. Therefore, when the DH line is used, the time and cost spent on fixing gene traits can be drastically reduced compared to the RIL group and the NIL group. In addition, He et al. (2001) created RIL and DH groups through “ZYQ8/JXI7” and then performed QTL mapping for major agricultural traits, and QTLs were mapped in the same area in the RIL group and the DH group. In addition, it was said that both the RIL group and the DH group were effective in performing QTL mapping. Some genes already discovered through QTL mapping were analyzed for expression through the CNDH line, and the functions of the genes were identified [26,27].

PCA analysis showed that BLB-susceptible lines were all distributed in the first and second quadrants. Moreover, BLB resistance lines were all distributed in four quadrants. In addition, when the phylogenetic tree was analyzed, the BLB resistance lines Nagdong, CNDH23, CNDH24, CNDH51, and CNDH54-1 all belonged to Group I. However, the susceptible lines CNDH69 and CNDH90 belonged to Group I, and Cheongcheong, CNDH81, and CNDH99 belonged to Group III. These results are consistent regarding the phenotype after infection with *Xoo* K3 strain for CNDH line in the field, wherein Cheongcheong, CNDH81, and CNDH99 completely died, but CNDH69 and CNDH90 showed moderate resistance rather than complete sensitivity. Therefore, CNDH69 and CNDH90 were sensitive but belonged to Group I. Moreover, it was reported that this type of inheritance is commonly reported in rice, according to a resistance study [28,29,30]. The completion of the marker-assisted rice genome sequencing and breeding has efficiently facilitated the discovery and mapping of QTLs and genes responsible for specific functions [31].

In the present research, we identified 28 target ORF regions on chromosome 6 that were associated with resistance to *Xoo* in the CNDH line. QTL analysis was carried out on the basis of phenotypic data evaluation. For transcription factor gene involved in BLB resistance identification, we used the CNDH line. The first-year QTL analysis identified four loci on three chromosomes, one on chromosome 6, two on chromosome 8, and one on chromosome 11. Compared with the second year and third year QTL data, the first-year data allowed for the identification of only one locus on chromosome number 6, which was flanked between marker RM20092 and RM20176, thereby being closely identical with the identified locus in the first-year analysis. Here, we postulated three main reasons as to why there is only one relevant interval for the QTL data of the second year. First, it is possible that multiple loci can simultaneously control the complex traits of a trait; second, the climate in the field test in the second year has changed too much from previous years (extreme heat); and third, there may be a certain measurement error when measuring the length of the infected leaf. However, despite these reasons, the overlap of the target regions between the markers RM20092 and RM20176 on chromosome 6 in the QTL data of the past two years showed that this similarity meant that the identified region is highly associated with transcription factor gene involved in BLB resistance. On the basis of the additive effects, we assumed that all the QTLs would show an inhibitory response toward BLB, because all the QTLs showed positive additive effects (Table S3). The phenotypic variation of all the QTLs ranged from 0.39% to 0.45%; however, other researchers reported a 31% phenotypic variation with 43.44 LOD value for the QTLs identified for BLB [32]. Countries such as China, South Korea, Japan, and Indonesia produce high quantities of rice and are severely affected by BLB, thereby reducing rice yield to a considerable amount. In the present study, we investigated the correlation of BLB lesion length and 1000 grain weight (Table S2). It was found that BLB infection significantly reduced grain weight, and the weight of grains further reduced as the BLB infection time increased. In order for the yield loss to be reduced in terms of weight decrease in infected plants, development of BLB-resistant lines is necessary. Therefore, the purpose of this study was to develop rice cultivars that are resistant to BLB, which will subsequently increase grain weight.

As a result of QTL analysis using the genetic map and central parameters (1000 grain weight and lesion length) of rice, it is possible to identify the genes responsible for BLB resistance, and in the process, increase the grain weight of rice. In the quantitative RT-PCR analyses of the target resistance gene *OsWRKYq6* in the resistant and susceptible group, it was found that the CNDH54-1 line in the resistant group had lower results than the other four resistant lines. Simultaneously, the resistance performance of CNDH54-1 in the collected phenotypic data was not the weakest. This indicated that there are other highly effective resistance mechanisms in CNDH54-1. In addition, OXCM was developed as a BLB resistance line, and when the relative expression level of *OsWRKYq6* was analyzed in OXCM, the expression level was similar to that of CNDH54-1, the strongest BLB resistance. WRKY transcription factor is widely involved in abiotic/biotic stress [33,34]. In particular, research on WRKY transcription factor is increasing rapidly, regardless of the type of plant at present. WRKY transcription factor is expressed when facing various stress environments and plants, being involved in the plant defense system by increasing the expression of PR (pathogen-related) proteins and signaling factors such as SA and JA [35]. In this study, QTLs related to BLB resistance were mapped. In addition, transcription factor genes involved in BLB resistance were screened in the mapped QTL region. *OsWRKYq6* is highly expressed in BLB-resistant lines. Therefore, *OsWRKYq6* is a transcription factor that is expected to be involved in BLB resistance and can be effectively used to conduct research related to BLB resistant in the future. We will conduct a targeted study on this matter in subsequent research. In the present study, further assessment associated with BLB resistance was not performed, but in the future, conducting follow-up studies that investigate how to overexpress all these ORF regions against *Xoo* may afford great benefit to rice-breeding countries.

We used QTL mapping to determine the interaction between genotype and phenotype, and a transcription factor called *OsWRKYq6* was found to be associated with the resistant phenotype. This gene was highly expressed in the BLB-resistant lines, and the sequence variation of *OsWRKYq6* was the same in Cheongcheong, Nagdong, CNDH51, and CNDH99 (Table S5). Although *OsWRKYq6* had the same sequence in both the resistant lines and the susceptible lines, only the resistant lines and the susceptible lines can be distinguished due to the difference in expression levels. To further confirm the gene function, researchers need to develop a mapping population in which this resistance gene is located on chromosome 6 in order to investigate its role in resistance to *Xoo* in rice.

## 4. Conclusions

BLB directly affects the decrease in rice yield. Various methods for screening resistant genes have been developed. In the present study, the BLB resistant transcription factor *OsWRKYq6* was screened from RM20092-RM20176 on chromosome 6 through field-investigated lesion length and genotype and interaction of 120 CNDH lines. *OsWRKYq6* has very high homology with the *WRKY* transcription factor. *OsWRKYq6* was highly expressed in BLB resistant lines after *Xoo* inoculation. However, the sequence of *OsWRKYq6* was the same in BLB resistant lines and BLB susceptible lines, and only due to the expression level, BLB resistant lines and BLB susceptible lines were distinguished. Additionally, to confirm the function of *OsWRKYq6*, it is necessary to analyze *Xoo* resistant through the development of a mapping population.5. Materials and Methods

### 4.1. Rice Materials and Field Design

The materials of Cheongcheong (IT228761, IT number is a resource number managed by the National Academy of Agricultural Sciences of Rural Development Administration, Jeonju, Korea)/Nagdong (IT006182) double haploid (CNDH) line was planted in the field at Kyungpook National University from 2010 [36]. F_1_ obtained through crossing of Cheongcheong (*Oryza sativa* spp. *indica* cv. Cheongcheong) and Nagdong (*Oryza sativa* spp. *japonica* cv. Nagdong) was cultured to double haploid, and CNDH 120 line was developed for genetic mapping. The CNDH 120 line was subjected to observational yield trials (OYT) and preliminary yield trials (PYT), a replicated yield trial (RYT), and a local adaptability test for 3 years. Local adaptability test in Daegu (Kyungpook National University, 41566, 80, Daehak-ro, Buk-gu, Daegu, Korea), Gunwi (Agricultural Education Center, 39061, 1610, Chisanhyoryeong-ro, Hyoryeong-myeon, Gunwi-gun, Gyeongsangbuk-do, Korea), and Jeonju (National Academy of Agricultural Science, Rural Development Administration, 55365, 166, Nongsaengmyeong-ro, Iseomyeon, WanjuGun, Jeollabuk-do, Korea). The 120 CNDH line was sterilized with seed sterilization solution and incubated for 4 days in darkness at 25 °C. Germinated seeds were sown in the fields of Kyungpook National University, Gunwi, and Jeonju, and the planting distance between plants was 30 × 15 cm. For each line, 125 plants were transplanted. The amount of fertilization was N–P_2_O_5_–K_2_O = 9-4.5-5.7 kg/10a according to the Agricultural Science and Technology Research Survey Standards of the Rural Development Administration.

### 4.2. K3 Strain Culture Media, Inoculation, and Infection Assessment

K3 strain of *Xoo* was cultured on potato sucrose agar media (PSA) containing 10 g of peptone, 10 g of sucrose, and 12 g of agar [37]. Prior to inoculation, the K3 strain was grown in liquid PSA for 72 h at 27 °C [38]. Leaf clipping was performed following the method previously described by Kauffman et al. [39]. The inoculation was started 40 days after the rice seedlings were transplanted. The middle 10 plants in each row were inoculated, and each plant was inoculated with five leaves. A pair of scissors was dipped into the K3 suspension, and then it was used to cut the leaf tip at approximately 3–4 cm to inoculate the bacterial strain. After the inoculation of the pathogenic strain, the length of the lesion of the infected leaf was measured after 14 days.

### 4.3. Genetic Map Construct and BLB Resistance QTL Mapping

The genetic map of the 120 CNDH line was constructed with 222 SSR markers using Mapmaker version 3.0 and the Kosambi mapping function. The map scanned 10.6 cM, resulting in average marker distance of 222 SSR markers. QTL analysis was performed using the Win QTL cart version 2.5 software, which was associated with the BLB lesion length of the CNDH line. In addition, a threshold LOD value of 3.0 or higher was used in Win QTL cart version 2.5 software [40]. The identified QTLs were named by following the method adapted by McCouch et al. [41].

### 4.4. Target Gene Selection and Physical Mapping

According to the results of QTL analysis, we can only obtain the associated regions in each chromosome on the gene map. On the basis of QTL data, we selected the target genes between the identified markers using the RICEXPRO, RAPDB, and NCBI databases and generated a physical map.

### 4.5. RNA Extraction

We used the RNeasy Plant Mini Kit (QIAGEN, Germany) to extract the total RNA from rice leaf samples at different time points (0 h, 1 h, 2 h, 4 h, 8 h, 16 h, 24 h, 48 h, 72 h) after inoculation. After sampling by inoculation time, the samples were immediately placed in liquid nitrogen and were manually ground using a mortar and pestle. According to the manufacturer’s instructions, the rice powder was suspended in 450 µL Buffer RLT with β-mercaptoethanol through the vortex method. The lysate was transferred to a QIAshredder spin column, centrifuged at 13,000 rpm for 1 min, and then transferred into a new tube. Next, 0.5 volume of (96–100%) ethanol was added. The mixture was moved into a RNeasy spin column (pink) and centrifuged for 15 s at 10,000 rpm to allow RNA binding within the spin column, and the residue was discarded. The column was washed by adding 700 µL Buffer RW1 and centrifuged for 1 min at 10,000 rpm. Finally, 500 µL Buffer RPE was used twice to remove any residual liquid in the spin column, followed by centrifugation for 1 and 2 min, respectively, at 10,000 rpm. RNA was eluted by adding 40 µL RNase-free water into a new collection tube up to a final volume of 1.5 mL, and then the mixture was centrifuged for 2 min at 13,000 rpm.

### 4.6. cDNA Synthesis

Diluted RNA from rice leaf samples at different time periods after inoculation was used to synthesize cDNA for quantitative RT-PCR analysis. The master mix was separately prepared for each part and treatment in the experiment. Then, 4 µL of 5× cDNA synthesis mix was added, including 10 µL of diluted RNA, 5 µL of RNase-free water, and 20× RTase (enzyme) in 1.5 mL tube. The master mix was allowed to incubate for 30 min at 42 °C. After incubation, cDNA concentration was measured using a NanoDrop 2000 spectrophotometer (Thermo Scientific, Wilmington, DE, USA).

### 4.7. Quantitative RT-PCR Analyses

We used the RNeasy plant mini kit (Qiagen, Germany), according to the manager’s instructions, and the RNA concentrations were measured using a NanoDrop 2000 spectro-photometer (Thermo Scientific, Wilmington, DE, USA). For first-strand cDNA synthesis, the qPCRBIO cDNA synthesis kit and 400 ng of the total RNA were used. Quantitative RT-PCR was performed using the Eco Real-Time PCR system (Illumina, Inc., San Diego, CA, USA), 2X qPCRBIO SyGreen (www.pcrbio.com, London, UK), and the specific primers for the examined gene expression. The *OsWRKYq6* gene sequence primers were used to determine *OsWRKYq6* (forward 5′-AGCAACAGGAGGTGCTTCAG-3′, reverse 5′-TCCCTGCTGGTAGGTAGTGG-3′). Among the CNDH lines, BLB resistance lines (OXCM, Nagdong, CNDH23, CNDH24, CNDH51, CNDH54-1) and susceptible lines (Cheongcheong, CNDH69, CNDH81, CNDH90, CNDH99) were compared with the expression level of OsWRKYq6 after inoculation of the K3 strain of *Xoo*. OXCM was provided by Jan et al. (2020) [19] and is a BLB-resistant line. The relative expression levels of *OsWRKYq6* were compared and analyzed in the OXCM and CNDH lines. Inoculation of the K3 strain of *Xoo* was conducted in the field (Daegu, Gunwi, Jeonju, Korea), and after inoculation, the phenotype was checked transferred to the pot (15 × 20).

### 4.8. Statistical Analysis

Using XLSTAT software, PCA (principal component analysis) and AHC (aggregation hierarchical clustering) statistical analysis modes were used to perform relevant statistical analysis on the three-year yield and length of lesions of rice leaves of the CNDH line. Five plants were used for each CNDH line, and the mean and standard deviation of lesion length, 1000 grain weight, and yield of five plants were statistically analyzed through SPSS [42].

## Data Availability

The data presented in this study are available on request from the corresponding author.

## Supplementary Materials

The following are available online at https://www.mdpi.com/article/10.3390/plants11030287/s1, Figure S1: Field inoculation experiment. Figure S2: The chromosomal location of QTL associated with lesion length and yield in CNDH genetic map for 3 years. Figure S3: Transcription factor gene involved in BLB resistance were screened from QTL. Figure S4: The BLB-resistant gene OsWRKYq6 amplification through PCR. Table S1: Distribution of lesion length and yield data after inoculated BLB to CNDH line over 3 years. Table S2: PCA statistical analysis of yield, 1000 grain weight, and lesion length data of CNDH line over three years. Table S3: Lesion length for QTL analysis result over 3 years. Table S4: The 28 related genes screened from the target interval of chromosome 6. Table S5: Detailed information of gene sequence of *OsWRKYq6*.
