# Supplementary material for: Genotype and Phenotype Interaction between OsWKRYq6 and BLB after Xanthomonas oryzae pv. Oryzae Inoculation in the Field
Source: Plants (Basel). 2022 Jan 21;11(3):287. doi: 10.3390/plants11030287 (PMC8840003; doi:10.3390/plants11030287)
Supplement: Supplementary file 1 [file plants-11-00287-s001.zip › Plants_supplementary materials_clear_2.pdf]

Supplementary Materials

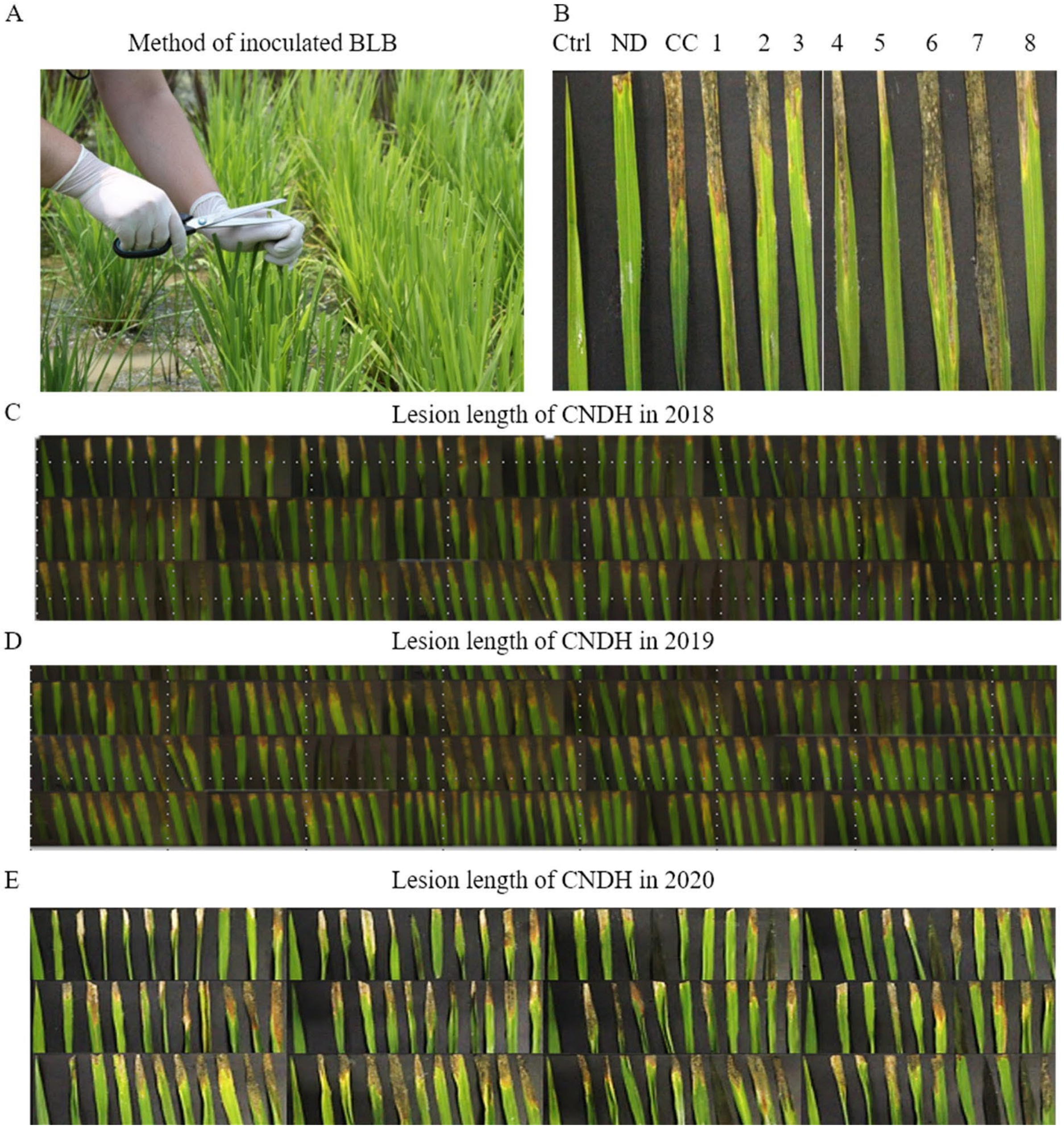

Figure S1. Field inoculation experiment. A: Scissor was dipped into the K3 suspension and then cut the related leaf tip about 3-4 cm that to inoculate bacterial strain; B: We found the leaf infection status of the control group, male parent and female parent (Cheongcheong/Nagdong) and CNDH populations of different lines after inoculation, as the disease progressed, the leaves turned yellow, turned into straw-colored and withered. At the same time, we found that the length of leaf infection fully reflects which

of the CNDH populations are highly resistant and those are weakly resistant.; C: The observation results of the length of leaf infection after inoculation of CNDH population in 2018; D: The observation results of the length of leaf infection after inoculation of CNDH population in 2019. E: The observation results of the length of leaf infection after inoculation of CNDH population in 2020.

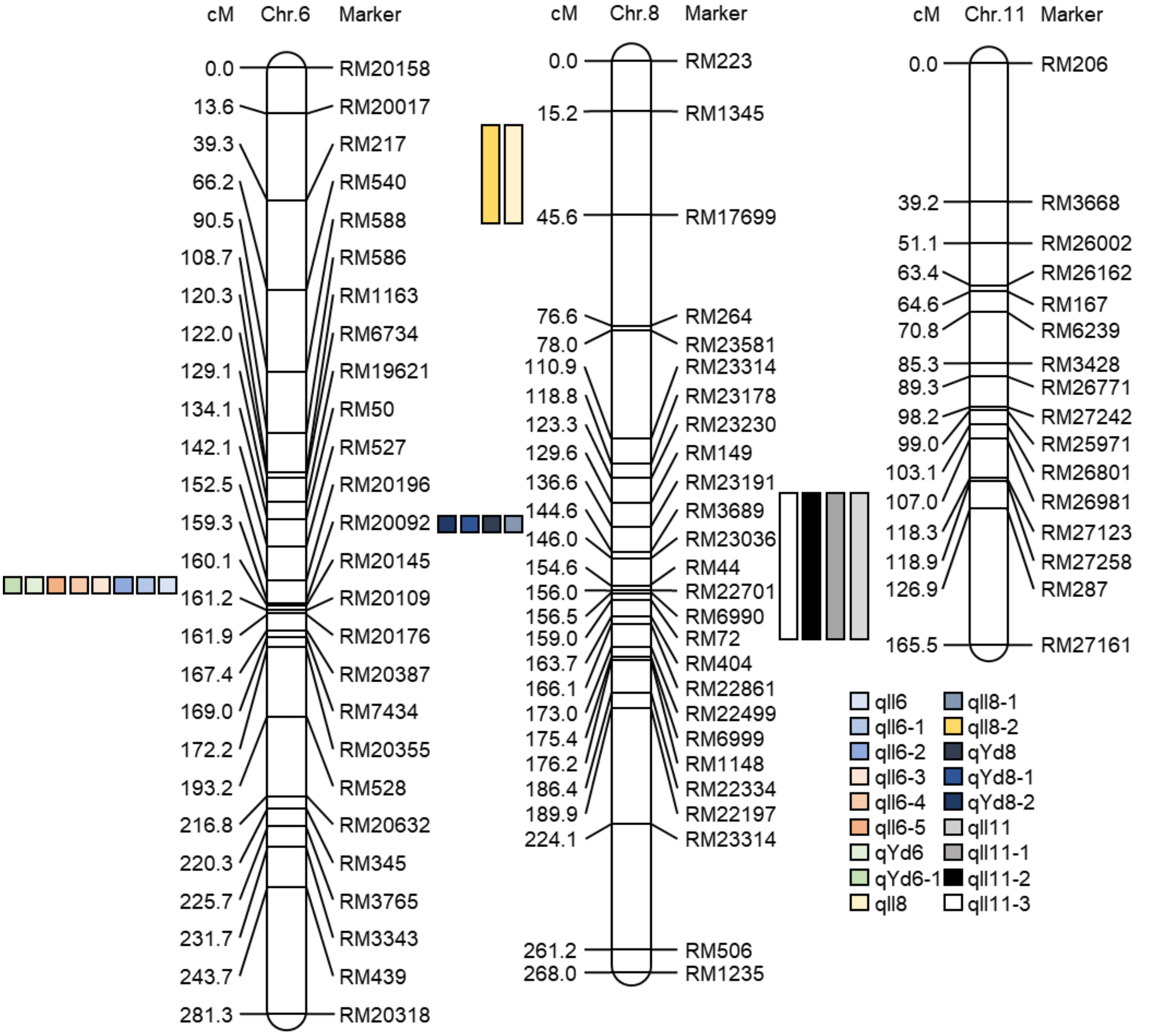

Figure S2. The chromosomal location of QTL associated with Lesion length and yield in CNDH genetic map for 3 years. The 8 different colored bars and logos in the lower left corner of this figure represent the target range obtained after QTL analysis of the length of infected leaves in the past two years. Among them, '*q*' means QTL, '*ll'* means the length of the infected leaf, 'Yd' means the yield of the plant and the last number represents the chromosome where the target region is located.

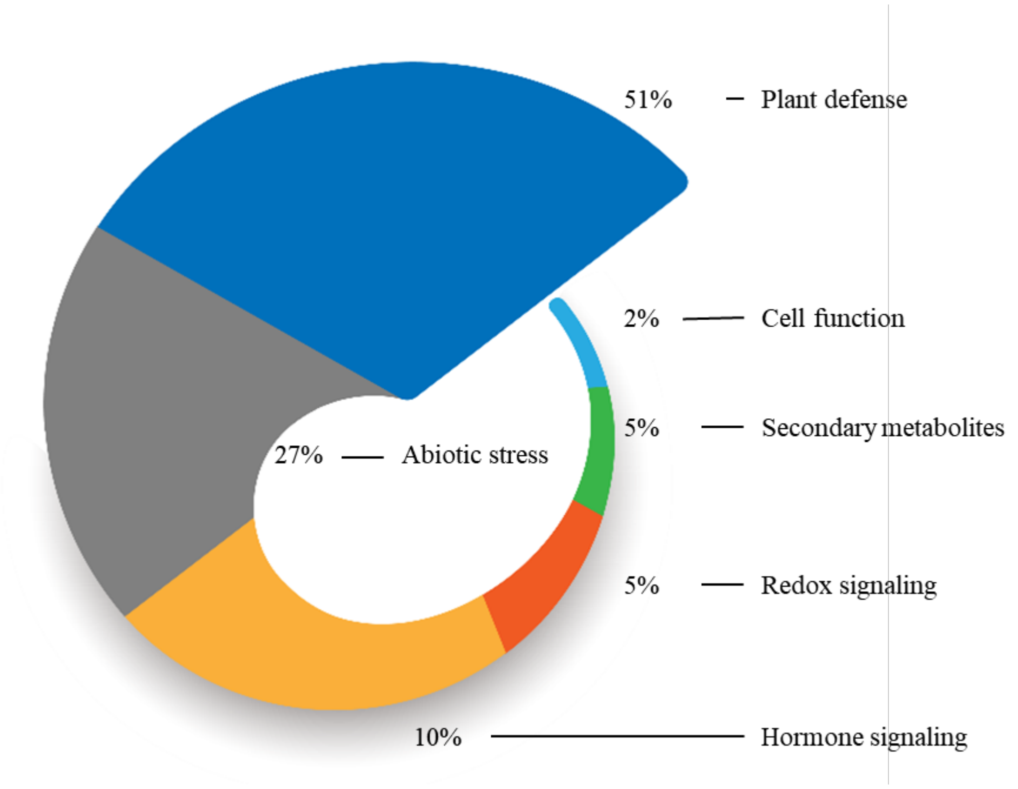

Figure S3. Transcription factor gene involved in BLB resistance between RM20092-RM20176 on chromosome 6. And classification by plant defense, redox signaling, abiotic stress, hormone signaling, cell function, secondary metabolites.

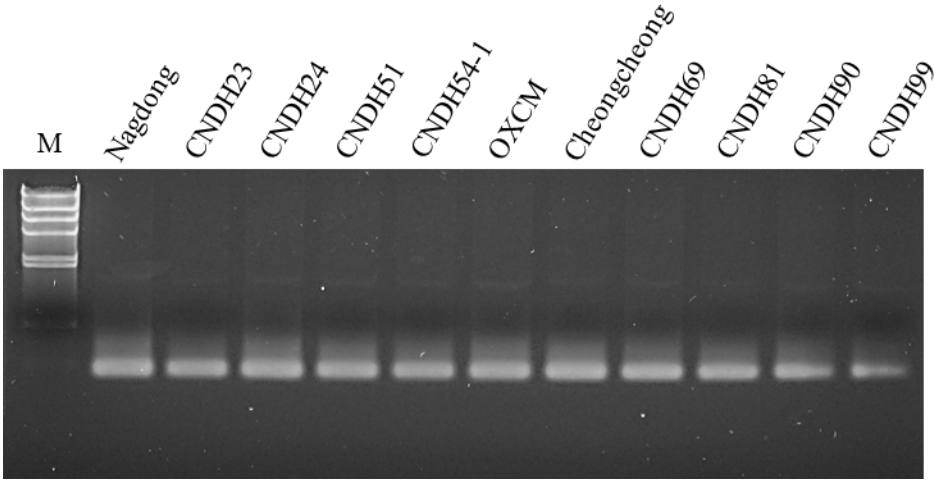

Figure S4. The BLB resistant gene OsWRKYq6 amplification through PCR. The OsWRKYq6 gene was amplified in the BLB resistant population and BLB susceptible population through PCR analysis. M; λ/*Hin*dIII DNA ladder, BLB resistant population; Nagdong, CNDH23, CNDH24, CNDH51, CNDH54-1, OXCM, BLB susceptible population; Cheongcheong, CNDH69, CNDH81, CNDH90, CNDH99.

Table S1. Distribution of lesion length and yield data after inoculated BLB to CNDH population in 3 years.

Year	Field test site	CNDH								
2018	Daegu	Lesion length (cm)	0.3-2.1	2.1-3.9	3.9-5.7	5.7-7.5	7.5-9.3	9.3-11.1	11.1-12.9	12.9-14.7
		Number of lines	18	21	23	25	14	6	4	4
		Yield(kg/10a)	0-60	60-120	120-180	180-240	240-300	300-360	360-420	420-480
		Number of lines	8	26	16	29	20	14	6	3
	Gunwi	Lesion length (cm)	0.5-2.5	2.5-4.5	4.5-6.5	6.5-8.5	8.5-10.5	10.5-12.5	12.5-14.5	14.5-16.5
		Number of lines	14	23	26	17	16	7	8	3
		Yield(kg/10a)	0-60	60-120	120-180	180-240	240-300	300-360	360-420	420-480
		Number of lines	16	22	30	19	24	7	3	1
	Jeonju	Lesion length (cm)	0.5-2.5	2.5-4.5	4.5-6.5	6.5-8.5	8.5-10.5	10.5-12.5	12.5-14.5	14.5-16.5
		Number of lines	21	15	21	27	11	8	7	3
		Yield(kg/10a)	0-60	60-120	120-180	180-240	240-300	300-360	360-420	420-480
		Number of lines	13	20	22	32	22	7	6	0
2019	Daegu	Lesion length (cm)	0.5-2.5	2.5-4.5	4.5-6.5	6.5-8.5	8.5-10.5	10.5-12.5	12.5-14.5	14.5-16.5
		Number of lines	21	17	20	24	14	9	6	4
		Yield(kg/10a)	0-60	60-120	120-180	180-240	240-300	300-360	360-420	420-480
		Number of lines	8	24	20	29	27	8	3	3
	Gunwi	Lesion length (cm)	0.5-2.5	2.5-4.5	4.5-6.5	6.5-8.5	8.5-10.5	10.5-12.5	12.5-14.5	14.5-16.5

		Number of lines	22	18	18	23	15	8	7	4
		Yield(kg/10a)	0-60	60-120	120-180	180-240	240-300	300-360	360-420	420-480
		Number of lines	13	20	19	32	23	7	6	3
	Jeonju	Lesion length (cm)	0.5-2.5	2.5-4.5	4.5-6.5	6.5-8.5	8.5-10.5	10.5-12.5	12.5-14.5	14.5-16.5
		Number of lines	22	17	19	23	15	9	6	4
		Yield(kg/10a)	0-60	60-120	120-180	180-240	240-300	300-360	360-420	420-480
		Number of lines	12	22	19	31	25	8	5	0
2020	Daegu	Lesion length (cm)	0.5-2.5	2.5-4.5	4.5-6.5	6.5-8.5	8.5-10.5	10.5-12.5	12.5-14.5	14.5-16.5
		Number of lines	18	12	28	21	14	9	1	1
		Yield(kg/10a)	0-60	60-120	120-180	180-240	240-300	300-360	360-420	420-480
		Number of lines	15	23	31	28	18	4	3	0
	Gunwi	Lesion length (cm)	0.5-2.5	2.5-4.5	4.5-6.5	6.5-8.5	8.5-10.5	10.5-12.5	12.5-14.5	14.5-16.5
		Number of lines	22	11	26	26	12	6	8	3
		Yield(kg/10a)	0-60	60-120	120-180	180-240	240-300	300-360	360-420	420-480
		Number of lines	15	24	27	32	15	6	3	0
	Jeonju	Lesion length (cm)	0.5-2.5	2.5-4.5	4.5-6.5	6.5-8.5	8.5-10.5	10.5-12.5	12.5-14.5	14.5-16.5
		Number of lines	23	16	20	23	15	8	6	4
		Yield(kg/10a)	0-60	60-120	120-180	180-240	240-300	300-360	360-420	420-480
		Number of lines	9	27	22	32	20	8	4	0

Table S2. PCA statistical analysis of yield, 1000-grain weight and lesion length data of CNDH population for three years.

	F1	F2	F3	F4	F5	F6	F7	F8	F9	F10
Y_2018_Control	0.184	0.102	0.503	0.064	-0.512	-0.105	0.046	0.364	0.183	0.239
Y_2018_Infected	0.326	-0.032	0.100	-0.271	-0.239	-0.412	-0.277	-0.262	-0.287	-0.397
Y_2019_Control	0.264	0.098	0.330	0.011	0.582	-0.020	0.410	-0.319	-0.057	-0.079
Y_2019_Infected	0.307	-0.072	0.072	-0.302	0.192	0.652	-0.320	0.122	0.133	0.197
Y_2020_Control	0.255	0.114	0.475	0.043	0.034	-0.071	0.258	0.029	0.073	0.092
Y_2020_Infected	0.340	-0.055	0.093	-0.307	-0.034	0.107	-0.320	-0.083	-0.091	-0.120
TGW_2018_Control	0.230	0.151	-0.074	0.542	-0.347	0.426	0.041	-0.370	-0.121	-0.140
TGW_2018_Infected	0.305	-0.083	-0.336	-0.102	-0.112	-0.237	0.086	-0.388	0.349	0.501
TGW_2019_Control	0.297	0.091	-0.144	0.323	0.386	-0.347	-0.346	0.392	0.045	0.057
TGW_2019_Infected	0.307	-0.114	-0.300	-0.127	-0.084	0.125	0.482	0.476	-0.202	-0.311
TGW_2020_Control	0.294	0.130	-0.124	0.465	0.064	-0.001	-0.191	0.056	-0.032	-0.034
TGW_2020_Infected	0.314	-0.101	-0.326	-0.117	-0.100	-0.060	0.288	0.039	0.079	0.103
LL_2018_Infected	-0.018	0.540	-0.112	-0.157	-0.016	0.017	0.000	0.000	0.588	-0.413
LL_2019_Infected	-0.020	0.539	-0.107	-0.170	-0.015	0.004	0.021	0.031	-0.563	0.406
LL_2020_Infected	-0.019	0.545	-0.111	-0.165	-0.015	0.010	0.011	0.016	-0.014	0.016

Table S3. Lesion length for QTL analysis result during 3 years.

Year	Traits	Location	QTLs	Interval Markers[z]	Chromosome	LOD	Additive effect[y]	R^{2}[x]	Increasing effects[w]
2018	Lesion length (cm)	Daegu	qll6	RM20092-RM20176	6	3.94	1.06	0.40	Cheongcheong
			qll8	RM1345-RM17699	8	3.09	1.66	0.44	Cheongcheong
			qll8-1	RM23178-RM23230	8	3.09	1.32	0.45	Cheongcheong
			qll11	RM27123-RM27161	11	3.30	0.99	0.41	Cheongcheong
		Gunwi	qll6-1	RM20092-RM20176	6	3.41	1.09	0.20	Cheongcheong
		Jeonju	qll6-2	RM20092-RM20176	6	3.81	1.08	0.25	Cheongcheong
			qll11-1	RM27123-RM27161	11	3.71	1.02	0.30	Cheongcheong
	Yield (kg/10a)	Daegu	qYd6	RM20092-RM20176	6	4.01	1.05	0.38	Cheongcheong
		Jeonju	qYd8	RM23178-RM23230	8	3.10	1.02	0.40	Cheongcheong
2019	Lesion length (cm)	Daegu	qll6-3	RM20092-RM20176	6	3.93	1.34	0.39	Cheongcheong
		Gunwi	qll11-2	RM27123-RM27161	11	3.01	1.28	0.32	Cheongcheong
		Jeonju	qll6-4	RM20092-RM20176	6	3.50	1.32	0.40	Cheongcheong
	Yield (kg/10a)	Jeonju	qYd8-1	RM23178-RM23230	8	3.05	1.00	0.20	Cheongcheong
2020	Lesion length (cm)	Daegu	qll6-5	RM20092-RM20176	6	2.6	1.03	0.37	Cheongcheong
			qll8-2	RM1345-RM17699	8	3.20	1.00	0.30	Cheongcheong
		Gunwi	qll11-3	RM26981-RM27161	11	2.88	1.12	0.38	Cheongcheong

Yield (kg/10a)	Daegu	qYd6-1	RM20092-RM20176	6		3.15	1.00		0.30	Cheongcheong
	Jeonju	qYd8-2	RM23178-RM23230	8		3.25	1.58		0.30	Cheongcheong

[z] Interval markers are those within the significance threshold on each border of the QTL range

[y] The proportion of evaluated phenotypic change attributable to a particular QTL was estimated using the coefficient of determination (R^2)

[x] Positive values of the additive effect indicate that alleles from Cheongcheong are in the direction of increasing the traits

[w] Increase allele is the source of the allele that causes an increase in the measured trait.

Table S4. The 28 related genes screened from the target interval of chromosome 6.

Gene	Gene locus	No. of genes
Cell function		17
Decapping 5	*Os06g0496000*	
Transcription factor bHLH83-like	*Os06g0496400*	
Retrotransposon unclassified	*Os06g0497600*	
Tyrosine specific phosphatase-like	*Os06g0498000*	
Alpha-glucan water dikinase 1 chloroplastic	*Os06g0498400*	
Nucleolar complex 3 homolog	*Os06g0498500*	
MOTHER of FT and TFL1 homolog 1-like	*Os06g0498800*	
GTP-binding BRASSINAZOLE INSENSITIVE PALE GREEN chloroplastic	*Os06g0498900*	
Pentatricopeptide repeat-containing At4g20090-like	*Os06g0499301*	
Iihydrolipoyllysine-residue acetyltransferase component 1 of pyruvate dehydrogena semitochondrial	*Os06g0499900*	
Proline-rich 2-like isoform X2	*Os06g0500300*	

Plant family expressed	*Os06g0502800*	
DGCR14	*Os06g0502900*	
Reticulon B2	*Os06g0503400*	
Probable CCR4-associated factor 1 homolog 9	*Os06g0503900*	
Agamous-like MADS-box AGL62	*Os06g0504100*	
HEAT repeat-containing 5B isoform X1	*Os06g0505100*	
Plant defense		2
ABC transporter G family member 4-like	*Os06g0503100*	
**WRKY transcription factor**	*OsWRKYq6*	
Redox signaling		5
Receptor kinase At4g00960	*Os06g0496800*	
Premnaspirodiene oxygenase-like	*Os06g0497200*	
Premnaspirodiene oxygenase-like	*Os06g0500700*	
Acyl-[acyl-carrier-] desaturase chloroplastic-like	*Os06g0503800*	

Stearoyl-acyl-carrier- desaturase4	*Os06g0503850*	
Abiotic stress		1
DNAJ heat shock N-terminal domain-containing	*Os06g0498450*	
Hormone signaling		2
Auxin-responsive GH3	*Os06g0499500*	
Indole-3-acetic acid-amido synthetase –like	*Os06g0499550*	
Secondary metabolites		1
Cytochrome P450 76M5-like	*Os06g0501900*	

Table S5. Detailed information of gene sequence of *OsWRKYq6*.

Line	BLB resistant/susceptible	Sequence (5' to 3')
Cheongcheong	Susceptible	ACCACCACTCCCACCCCCCCCCTCTCTCTCTCTACCTACCTTTCTCTCTCTAGCAAAATACTAAGCTCAA
		AGTCAGCGCCCTAGCTAGTTTCTCCGAGGCGGCGAGATGGACGGCGACGCATGGTGGTACCCTGGCGGCG
		GCGGAGGTGGTGGTAGCAACAACTGGGATCTTGGCGCTGTCGTGAGGTTTGGGTGCGGCGGGGGACGGGT
		GAGCCCGGCGGCGGCGTTGCTGGGCGAGGCGTGGGAGTACGACGACGACCCGTTCTCGTCGTTCCTCGCG
		CCGCCCATGACGGCGCAGCAGGCGGCGCTGCCCGCCGTGTGGGAGGAGGGAGACGACGGGGACGCGGCGT
		GGATGGCGCCGCTCCCGGGGCTTCAGACCGGTGGTGGCTGGGGTGATCAGGCCCCCATGGTCGTCGACGA
		GCTCTGCGGGGCCCTCGTTGTGGCCCCTCCTCCACCTCCGAAGCAACAGGAGGTGCTTCAGGTCCAGCAG
		CAGCCGCCGCCGGCGGACAACACGCAGCCCACTACCTACCAGCAGGGATCCGGCGGCGACGGTGAGAGCA
		CTCGCGCCGGCGGATCAAGGTCGGCGAGGAGAAAGAAGAAGCAGACGAGAAAGGAGGTGGTGCGCGTGGC
		GGCGAGCGGGCCGGCGCCGGACTTGTGGGCGTGGCGGAAGTACGGGCAGAAGCCGATCAAGGGGTCCCCG
		TACCCGCGCGGCTACTACCGGTGCAGCAGCAACAAGAACTGCGCCGCCCGGAAGCAGGTGGAGCGCTGCC
		GCTTCGACCCCTCCTTCCTCCTCCTCACCTACACCGGCGCCCACTCCGGCCACGACGTCCCCCTCCACCG
		CAACTCCCTCGCCGGCACCACGCGCCACAAGCCGCCGCCGCCGCCGCTCCCGTCCGCCGCCGACAAG
		TCTCCGGCCACCGCGGCTGAGGCGGCGACGGCGAGCCAGAGCCCCGGCCTGTCGCCGACCACGCCGCTGC
		GCGCCTCGTCCATGGAGCTCCACGGCGAGGACGACGCCGAGGCCGAGCTGCAGGTGGAGGAGGACGACAT
		GGCCATCGACGACGAAGACGACGACGACGTCGCCGACGAGACCATCAGCACCGTGCCGTGGGGCACGCCC
		ATCTCCGACGCGATCATCGCGGCGAGCTACGAGTGGAGATGACCACCACCACCTCGGCGACCTCGCCGTA
		CATTGCCATACATAGCAAGCAATTCAGCCGTCTTAGAGAGAGAAATTAAGGGAAAGAGACAGAAATTAAA
		GAGAGAGTTGAAGGGAGCATCCGATCCATGCAGCTAGTATCTCCAACACCCAGTAGCACTAGTGTTTAAT
		TACTTAATTAATTACTACAAGCACCTAGTAGCGTCTATTTACAGAACTACCTCCGTTCCAAAATAAGTGC
		AGTCGTGAATATCCGTACCCAACGTTTGACCGTCCGTCTTATTTGAAAAATTTGTGAAAAATTTGAAAAT
		A
Nagdong	Resistant	ACCACCACTCCCACCCCCCCCCTCTCTCTCTCTACCTACCTTTCTCTCTCTAGCAAAATACTAAGCTCAA
		AGTCAGCGCCCTAGCTAGTTTCTCCGAGGCGGCGAGATGGACGGCGACGCATGGTGGTACCCTGGCGGCG
		GCGGAGGTGGTGGTAGCAACAACTGGGATCTTGGCGCTGTCGTGAGGTTTGGGTGCGGCGGGGGACGGGT

		GAGCCCGGCGGCGGCGTTGCTGGGCGAGGCGTGGGAGTACGACGACGACCCGTTCTCGTCGTTCCTCGCG<br>CCGCCCATGACGGCGCAGCAGGCGGCGCTGCCCGCCGTGTGGGAGGAGGGAGACGACGGGGACGCGGCGT<br>GGATGGCGCCGCTCCCGGGGCTTCAGACCGGTGGTGGCTGGGGTGATCAGGCCCCCATGGTCGTCGACGA<br>GCTCTGCGGGGCCCTCGTTGTGGCCCCTCCTCCACCTCCGAAGCAACAGGAGGTGCTTCAGGTCCAGCAG<br>CAGCCGCCGCCGGCGGACAACACGCAGCCCACTACCTACCAGCAGGGATCCGGCGGCGACGGTGAGAGCA<br>CTCGCGCCGGCGGATCAAGGTCGGCGAGGAGAAAGAAGAAGCAGACGAGAAAGGAGGTGGTGCGCGTGGC<br>GGCGAGCGGGCCGGCGCCGGACTTGTGGGCGTGGCGGAAGTACGGGCAGAAGCCGATCAAGGGGTCCCCG<br>TACCCGCGCGGCTACTACCGGTGCAGCAGCAACAAGAACTGCGCCGCCCGGAAGCAGGTGGAGCGCTGCC<br>GCTTCGACCCCTCCTTCCTCCTCCTCACCTACACCGGCGCCCACTCCGGCCACGACGTCCCCCTCCACCG<br>CAACTCCCTCGCCGGCACCACGCGCCACAAGCCGCCGCCGCCGCCGCTCCCGTCCGCCGCCGACAAG<br>TCTCCGGCCACCGCGGCTGAGGCGGCGACGGCGAGCCAGAGCCCCGGCCTGTCGCCGACCACGCCGCTGC<br>GCGCCTCGTCCATGGAGCTCCACGGCGAGGACGACGCCGAGGCCGAGCTGCAGGTGGAGGAGGACGACAT<br>GGCCATCGACGACGAAGACGACGACGACGTCGCCGACGAGACCATCAGCACCGTGCCGTGGGCACGCCC<br>ATCTCCGACGCGATCATCGCGGCGAGCTACGAGTGGAGATGACCACCACCACCTCGGCGACCTCGCCGTA<br>CATTGCCATACATAGCAAGCAATTCAGCCGTCTTAGAGAGAGAAATTAAGGGAAAGAGACAGAAATTAAA<br>GAGAGAGTTGAAGGGAGCATCCGATCCATGCAGCTAGTATCTCCAACACCCAGTAGCACTAGTGTTTAAT<br>TACTTAATTAATTACTACAAGCACCTAGTAGCGTCTATTTACAGAACTACCTCCGTTCCAAAATAAGTGC<br>AGTCGTGAATATCCGTACCCAACGTTTGACCGTCCGTCTTATTTGAAAAATTTGTGAAAAATTTGAAAAT<br>A
CNDH51	Resistant	ACCACCACTCCCACCCCCCCCCTCTCTCTCTCTACCTACCTTTCTCTCTCTAGCAAAATACTAAGCTCAA<br>AGTCAGCGCCCTAGCTAGTTTCTCCGAGGCGGCGAGATGGACGGCGACGCATGGTGGTACCCTGGCGGCG<br>GCGGAGGTGGTGGTAGCAACAACTGGGATCTTGGCGCTGTCGTGAGGTTTGGGTGCGGCGGGGGACGGGT<br>GAGCCCGGCGGCGGCGTTGCTGGGCGAGGCGTGGGAGTACGACGACGACCCGTTCTCGTCGTTCCTCGCG<br>CCGCCCATGACGGCGCAGCAGGCGGCGCTGCCCGCCGTGTGGGAGGAGGGAGACGACGGGGACGCGGCGT<br>GGATGGCGCCGCTCCCGGGGCTTCAGACCGGTGGTGGCTGGGGTGATCAGGCCCCCATGGTCGTCGACGA<br>GCTCTGCGGGGCCCTCGTTGTGGCCCCTCCTCCACCTCCGAAGCAACAGGAGGTGCTTCAGGTCCAGCAG<br>CAGCCGCCGCCGGCGGACAACACGCAGCCCACTACCTACCAGCAGGGATCCGGCGGCGACGGTGAGAGCA<br>CTCGCGCCGGCGGATCAAGGTCGGCGAGGAGAAAGAAGAAGCAGACGAGAAAGGAGGTGGTGCGCGTGGC

CNDH99	Susceptible	GGCGAGCGGGCCGGCGCCGGACTTGTGGGCGTGGCGGAAGTACGGGCAGAAGCCGATCAAGGGGTCCCCG<br>TACCCGCGCGGCTACTACCGGTGCAGCAGCAACAAGAACTGCGCCGCCCGGAAGCAGGTGGAGCGCTGCC<br>GCTTCGACCCCTCCTTCCTCCTCCTCACCTACACCGGCGCCCACTCCGGCCACGACGTCCCCCTCCACCG<br>CAACTCCCTCGCCGGCACCACGCGCCACAAGCCGCCGCCGCCGCCGCTCCCGTCCGCCGCCGACAAG<br>TCTCCGGCCACCGCGGCTGAGGCGGCGACGGCGAGCCAGAGCCCCGGCCTGTCGCCGACCACGCCGCTGC<br>GCGCCTCGTCCATGGAGCTCCACGGCGAGGACGACGCCGAGGCCGAGCTGCAGGTGGAGGAGGACGACAT<br>GGCCATCGACGACGAAGACGACGACGACGTCGCCGACGAGACCATCAGCACCGTGCCGTGGGCACGCCC<br>ATCTCCGACGCGATCATCGCGGCGAGCTACGAGTGGAGATGACCACCACCACCTCGGCGACCTCGCCGTA<br>CATTGCCATACATAGCAAGCAATTCAGCCGTCTTAGAGAGAGAAATTAAGGGAAAGAGACAGAAATTAAA<br>GAGAGAGTTGAAGGGAGCATCCGATCCATGCAGCTAGTATCTCCAACACCCAGTAGCACTAGTGTTTAAT<br>TACTTAATTAATTACTACAAGCACCTAGTAGCGTCTATTTACAGAACTACCTCCGTTCCAAAATAAGTGC<br>AGTCGTGAATATCCGTACCCAACGTTTGACCGTCCGTCTTATTTGAAAAATTTGTGAAAAATTTGAAAAT<br>A<br>ACCACCACTCCCACCCCCCCCCTCTCTCTCTCTACCTACCTTTCTCTCTCTAGCAAAATACTAAGCTCAA<br>AGTCAGCGCCCTAGCTAGTTTCTCCGAGGCGGCGAGATGGACGGCGACGCATGGTGGTACCCTGGCGGCG<br>GCGGAGGTGGTGGTAGCAACAACTGGGATCTTGGCGCTGTCGTGAGGTTTGGGTGCGGCGGGGGACGGGT<br>GAGCCCGGCGGCGGCGTTGCTGGGCGAGGCGTGGGAGTACGACGACGACCCGTTCTCGTCGTTCCTCGCG<br>CCGCCCATGACGGCGCAGCAGGCGGCGCTGCCCGCCGTGTGGGAGGAGGGAGACGACGGGGACGCGGCGT<br>GGATGGCGCCGCTCCCGGGGCTTCAGACCGGTGGTGGCTGGGGTGATCAGGCCCCCATGGTCGTCGACGA<br>GCTCTGCGGGGCCCTCGTTGTGGCCCCTCCTCCACCTCCGAAGCAACAGGAGGTGCTTCAGGTCCAGCAG<br>CAGCCGCCGCCGGCGGACAACACGCAGCCCACTACCTACCAGCAGGGATCCGGCGGCGACGGTGAGAGCA<br>CTCGCGCCGGCGGATCAAGGTCGGCGAGGAGAAAGAAGAAGCAGACGAGAAAGGAGGTGGTGCGCGTGGC<br>GGCGAGCGGGCCGGCGCCGGACTTGTGGGCGTGGCGGAAGTACGGGCAGAAGCCGATCAAGGGGTCCCCG<br>TACCCGCGCGGCTACTACCGGTGCAGCAGCAACAAGAACTGCGCCGCCCGGAAGCAGGTGGAGCGCTGCC<br>GCTTCGACCCCTCCTTCCTCCTCCTCACCTACACCGGCGCCCACTCCGGCCACGACGTCCCCCTCCACCG<br>CAACTCCCTCGCCGGCACCACGCGCCACAAGCCGCCGCCGCCGCCGCTCCCGTCCGCCGCCGACAAG<br>TCTCCGGCCACCGCGGCTGAGGCGGCGACGGCGAGCCAGAGCCCCGGCCTGTCGCCGACCACGCCGCTGC<br>GCGCCTCGTCCATGGAGCTCCACGGCGAGGACGACGCCGAGGCCGAGCTGCAGGTGGAGGAGGACGACAT

	GGCCATCGACGACGAAGACGACGACGACGTCGCCGACGAGACCATCAGCACCGTGCCGTGGGGCACGCCC<br>ATCTCCGACGCGATCATCGCGGCGAGCTACGAGTGGAGATGACCACCACCACCTCGGCGACCTCGCCGTA<br>CATTGCCATACATAGCAAGCAATTCAGCCGTCTTAGAGAGAGAAATTAAGGGAAAGAGACAGAAATTAAA<br>GAGAGAGTTGAAGGGAGCATCCGATCCATGCAGCTAGTATCTCCAACACCCAGTAGCACTAGTGTTTAAT<br>TACTTAATTAATTACTACAAGCACCTAGTAGCGTCTATTTACAGAACTACCTCCGTTCCAAAATAAGTGC<br>AGTCGTGAATATCCGTACCCAACGTTTGACCGTCCGTCTTATTTGAAAAATTTGTGAAAAATTTGAAAAT<br>A
